# Our Premium

**Published:** 1879-01-20

**Authors:** 


### ☞ *OUR PREMIUM.* ☜

C. HENRI LEONARD'S *Day Book* is offered as a premium for two new subscribers to the *Record*. It is most convenient and valuable for the practitioner. See Leonard's advertisement.

---

Dr. H. P. BABCOCK, of Oakland, California, died on 27th ult.

